# Real-world diagnostic performance of artificial intelligence in skin cancer screening

**DOI:** 10.1093/skinhd/vzag068

**Published:** 2026-06-09

**Authors:** Charles H Earnshaw, Karmen Cheung, Fasiha Arshad, Ross Moore, Ali Al-Janabi, Emma McMullen

**Affiliations:** Dermatology Centre, Northern Care Alliance NHS Foundation Trust & Division of Musculoskeletal and Dermatological Sciences, Manchester NIHR Biomedical Research Centre, Manchester Academic Health Science Centre, University of Manchester, Manchester,UK; Cancer Research UK Manchester Institute, University of Manchester, Manchester,UK; Department of Dermatology, Salford Royal Hospital, Northern Care Alliance NHS Foundation Trust, Manchester, UK; Department of Dermatology, Salford Royal Hospital, Northern Care Alliance NHS Foundation Trust, Manchester, UK; Department of Dermatology, Salford Royal Hospital, Northern Care Alliance NHS Foundation Trust, Manchester, UK; Dermatology Centre, Northern Care Alliance NHS Foundation Trust & Division of Musculoskeletal and Dermatological Sciences, Manchester NIHR Biomedical Research Centre, Manchester Academic Health Science Centre, University of Manchester, Manchester,UK; Department of Dermatology, Salford Royal Hospital, Northern Care Alliance NHS Foundation Trust, Manchester, UK; Greater Manchester Skin Pathway Board, Greater Manchester Integrated Care Board, Manchester, UK

## Abstract

**Background:**

The use of artificial intelligence (AI) in many aspects of society is expanding rapidly. AI has recently been employed in the National Health Service skin cancer referral pathway. We sought to assess the real-world diagnostic performance of AI in this setting.

**Objectives:**

To assess the real-world diagnostic performance, at the histopathological level, of AI employed in the urgent skin cancer screening pathway.

**Methods:**

This was a prospective observational study of the first 3 months of skin cancer referrals assessed by the Deep Ensemble for Recognition of Malignancy (DERM) AI algorithm in a tertiary care dermatology department in the North West of England. All lesions assessed by the algorithm were included in the analysis. Participant data were retrieved from medical records. Outcomes assessed included the AI diagnosis, whether a human review of AI diagnosis occurred, the face-to-face dermatologist’s diagnosis and the outcome of the dermatologist’s assessment. Comparison was made particularly between the final histopathological diagnosis, and AI and dermatologist diagnoses.

**Results:**

AI had a sensitivity of 95.3% [95% confidence interval (CI) 90.5–98.1], which compared favourably with dermatologists (88.5%, 95% CI 82.3–93.2; *P* = 0.006). The positive predictive value of the AI algorithm was lower, at 46.5% (95% CI 44.6–48.5). This compared with 62.1% (95% CI 53.9–67.7) in dermatologists. A total of 318 AI assessments with no remote human review went on to have their lesions reviewed by a dermatologist and biopsied. AI correctly identified the precise diagnosis 28.6% of the time, compared with dermatologists 61.6% of the time (*P* < 0.001). The correct tumour/lesion type was identified by AI 51.4% of the time and by dermatologists 75.5% (*P* < 0.001). In lesions that the AI deemed benign, and that would have been discharged with no human review, four cancers were diagnosed.

**Conclusions:**

AI has high sensitivity in the detection of skin cancer. However, the diagnostic accuracy of the information provided by AI to clinicians is low and could be further optimized to reduce the risk of automation bias. Furthermore, this study suggests the removal of human validation of AI decisions may be premature due to the potential for missed cancer diagnoses.

## Teaser

We sought to investigate the diagnostic performance at the histopathological level of a newly deployed artificial intelligence (AI) algorithm used in the screening of suspected skin cancer referrals. We surprisingly found that the diagnosis presented to the dermatologist by AI was correct 28.6% of the time. This finding highlights the importance of dermatologists not relying solely on information presented by AI due to the risk of automation bias.

What is already known about this topic?

- Many published studies show the high accuracy of artificial intelligence (AI) in the classification of lesions referred as potential skin cancers.
- However, the postimplementation performance of AI in clinical settings has not been fully explored, and there is a demand for this information.
- This study comes at a critical time due to an increasing number of hospitals considering autonomous use of the AI algorithm.

What does this study add?

- This study suggests that the real-world diagnostic performance of AI, as confirmed by the underlying histopathological diagnosis, remains below the level of dermatologists.
- The findings suggest that human review of AI decisions still has value in preventing missed cancer diagnoses, and that optimal diagnostic ability is achieved when combining AI and clinician assessments.

Skin cancers are the most common form of human malignancy.^1^ Melanoma represents the fifth most common form of cancer, and despite recent progress in the treatment of advanced melanoma,^2,3^ remains the deadliest form of skin cancer.^4^ Cutaneous squamous cell carcinoma (cSCC) has a higher than traditionally thought rate of metastasis,^1,5^ and is forecast to overtake melanoma mortality in the UK.^6^ Together, this high burden of disease means suspected skin cancer referrals make up half of outpatient dermatology referrals.^7^

Artificial intelligence (AI) has the potential to influence many aspects of society, and its use is rapidly being adopted in numerous sectors, including healthcare. Earlier work in 2017 and 2018 showed the promise that AI algorithms (in this context, convolutional neural networks) have in delivering dermatologist-level diagnostic ability.^8,9^ AI-based tools building on this technology have recently been approved for use autonomously within clinical settings in the UK.^10^ The accurate triage and diagnosis of skin cancer by AI could be of major value in relieving clinical pressures from overstretched dermatology departments^11^ and potentially in reducing waiting times.

It has been claimed through the assessment of multiple AI algorithms that their performance approaches, or even exceeds, that of trained dermatologists.^8,9,12–14^ However, further elucidation of AI product accuracy for tools now implemented in clinical practice is a critical unmet need.^10,15–17^ Moreover, what has not yet been fully assessed is the real-world accuracy of AI lesion classification in the clinical setting in direct comparison with the underlying histopathological diagnosis. We therefore sought to assess the accuracy, at the critical definitive histopathological level,^18^ of the AI diagnosis communicated to dermatologists in comparison with the diagnosis of trained dermatologists. We also focused on whether the approval for AI to act autonomously in skin cancer screening^10^ has the potential to result in missed cancer diagnoses.

## Patients and methods

### Study design

In this prospective observational study of patients with suspected skin cancer referred to a tertiary care dermatology department in the North West of England, the first 3 months of suspected skin cancer referrals assessed using the Deep Ensemble for Recognition of Malignancy (DERM) AI algorithm were analysed (1230 lesions in total). This algorithm has high sensitivity for the detection of melanoma, and takes into account high-resolution lesion images, dermoscopy and the patient’s clinical history.^19^

**Figure 1:**
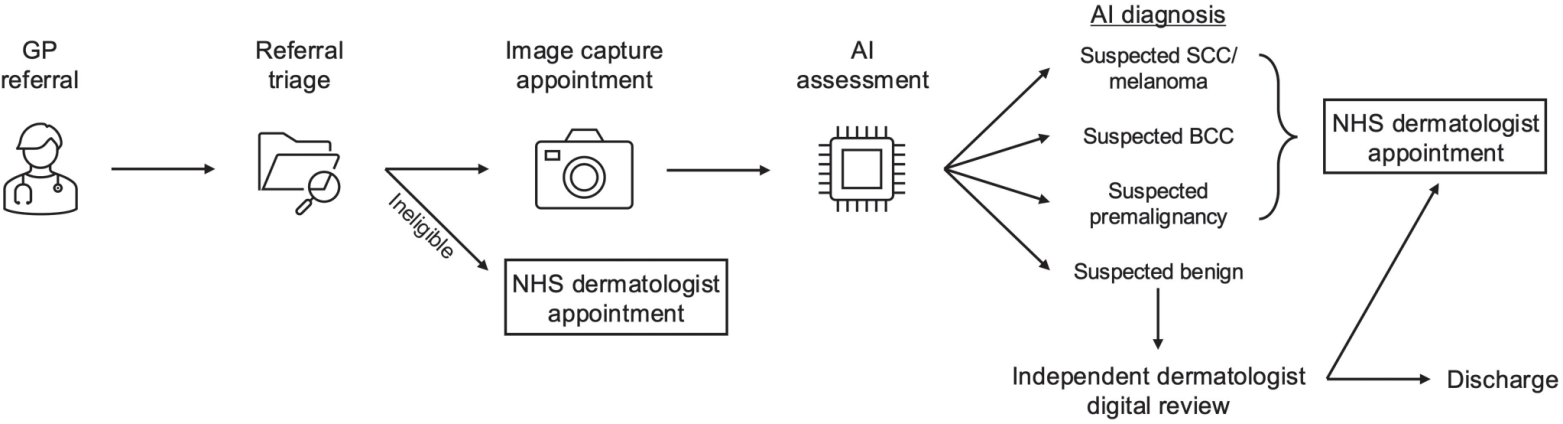
Suspected skin cancer patient pathway. Flow diagram illustrating the artificial intelligence (AI)-assisted skin-cancer screening pathway assessed in this study. BCC, basal cell carcinoma; GP, general practitioner; NHS, National Health Service; SCC, squamous cell carcinoma.

To reduce bias, all patients assessed via the algorithm were included in this study. Figure 1 shows the patient pathway, from general practitioner (GP) referral to dermatologist assessment.

There are several reasons why referrals may be ineligible for assessment by AI. Patients younger than 18 years of age, and complex lesions, including ulcerated lesions, large lesions, lesions obscured by hair, tattoos and scars, lesions on the mucosa, genital and palmoplantar surfaces, and previously biopsied lesions, are not eligible for assessment via AI. Additionally, patients with four or more lesions, and lesions >2.3 cm, are not eligible for AI assessment. Therefore, the population included in this study is slightly different from the background population; however, it represents a full analysis of the AI-assessed suspected patients with skin cancer in our Trust. Referrals were excluded from analysis if the AI report was not attached on the system despite having image-capture appointments, were duplicates or were from previously biopsied or incompletely excised tumours.

### Outcomes

Data regarding the GP’s suspected diagnosis, AI algorithm diagnosis presented to subsequently reviewing clinicians, presence or not of a human review of the AI output, the subsequent dermatologist diagnosis and plan, and histopathological diagnosis were collected from medical records by the clinical care team and anonymized before analysis. AI algorithm outcome depended on the suspected diagnosis (Figure 1). Gender and ethnicity data were automatically collected from patient records: these data were provided by the patient upon registration at their first appointment.

For assessment of diagnostic accuracy of the AI algorithm, only cases in which the AI algorithm did not undergo a human review of the images were taken forward for comparison with the histopathological diagnosis (representing 711 total cases). This ensured the most direct possible comparison of AI diagnosis, in-person dermatologist diagnosis and histopathological diagnosis. This does not, therefore, capture full sensitivity and specificity information of the AI algorithm acting autonomously, because all potential discharges by the AI algorithm in our Trust automatically undergo a human review to validate them. A number of these AI-planned discharges were overturned, which allows for further subgroup analysis as presented in the results section.

Outcomes included whether a skin cancer was diagnosed, and whether the AI algorithm or dermatologist thought skin cancer was present. Additional outcomes included whether the AI or dermatologist diagnosis was precisely correct (e.g. a differential diagnosis of melanoma that turned out to be a dysplastic naevus was incorrect), or whether the AI or dermatologist diagnosis was within the correct cell lineage or tumour type (in which case the previous example would be scored as correct).

### Statistical analysis

We included all patients assessed by the DERM AI algorithm during the first 3 months of its use. Statistical significance was assessed with a two-tailed McNemar’s test using Prism version 10 (GraphPad, La Jolla, CA, USA). Sensitivity, specificity, positive predictive value (PPV) and negative predictive value (NPV) were calculated using MedCalc’s online calculator.^20^ Only cases in which the AI lesion classification [possible diagnoses: melanoma, atypical naevus, benign, SCC, SCC *in situ*, actinic keratosis (AK) lesions, basal cell carcinoma (BCC)] was visible to the dermatologist were included for calculation of sensitivity, specificity, PPV and NPV (i.e. lesions in which the AI diagnosis was blank or unable to be generated due to poor image quality were excluded). This resulted in the assessment of a total of 353 lesions with final histopathology [318 in cases with no independent dermatologist review of AI decision before a National Health Service (NHS) appointment, with a further 35 performed in cases in which there was an independent dermatologist review of AI triage before clinical assessment]. Data were prepared for publication using Microsoft Office, Prism version 10 and SankeyMATIC.com.

## Results

Across the first 3 months of AI use in our Trust, commencing in November 2024, 1230 suspected skin cancer lesion referrals were identified by our audit as being assessed by the AI algorithm. The baseline characteristics of the patients with these lesions are shown in Table 1, and the suspected skin cancer referral pathway in Figure 1.

**Table 1:** Baseline patient characteristics

	*n* (%)
Sex	
Female	726 (59.0)
Male	504 (41.0)
Age (years), mean (IQR)	
Female	55.18 (41–69)
Male	61.55 (51–75)
Ethnicity	
White (British)	971 (78.9)
White (Other)	33 (2.7)
Asian	16 (1.3)
African	4 (0.3)
Other^a^	11 (0.9)
Unknown/declined	195 (15.9)
GP referral diagnosis	
Melanoma	511 (41.5)
Squamous cell carcinoma	498 (40.5)
Basal cell carcinoma	188 (15.3)
Other/not stated	33 (2.7)

Population characteristics of the 1230 lesions assessed in this study. GP, general practitioner; IQR, interquartile range. ^a^The ‘Other’ classification encompassed ‘Any other ethnic group’ as recorded by the clinical system.

**Figure 2:**
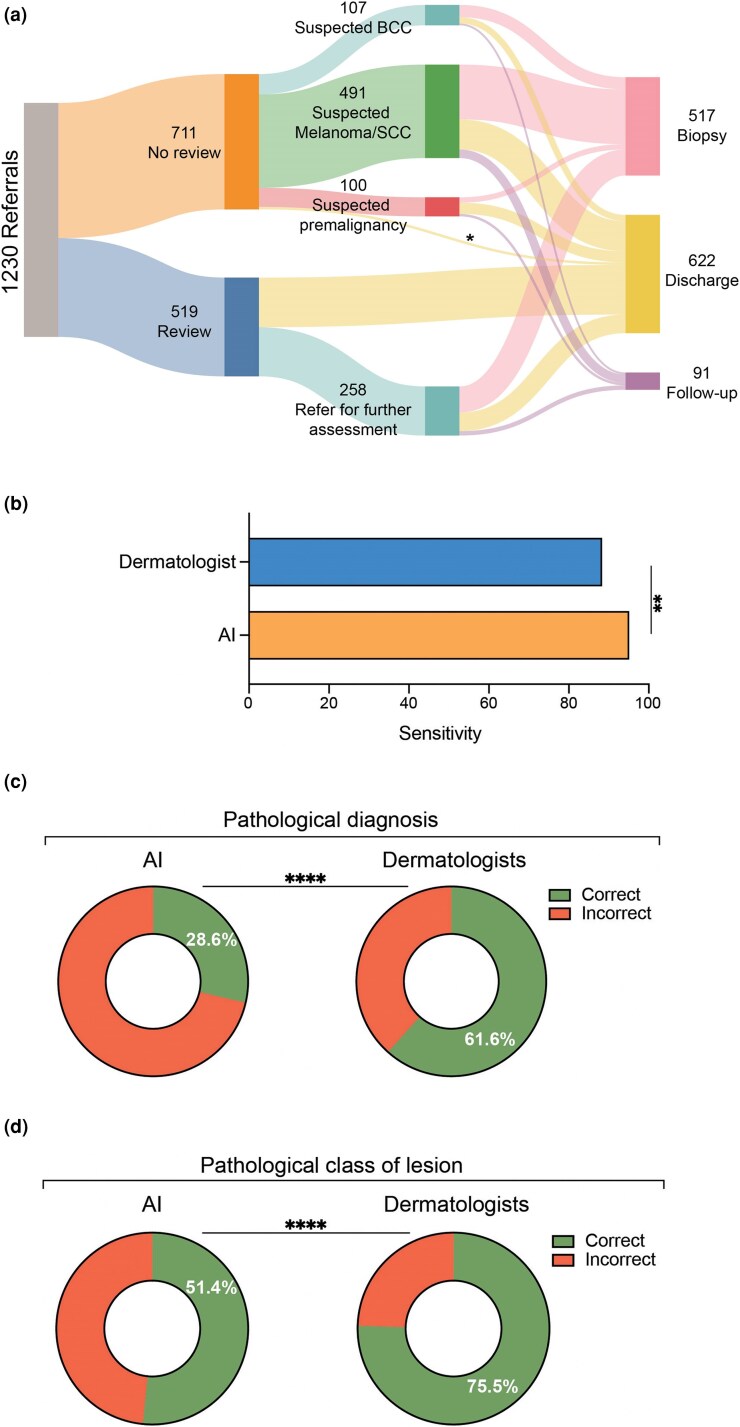
Dermatologists outperform artificial intelligence (AI) at predicting the correct pathological diagnosis. (a) Sankey diagram illustrating the clinical pathway of suspected skin cancer lesions assessed by AI and examined by our study. *Indicates a small group of lesions that would have been discharged from the ‘no review’ arm; however, as the patients had an additional, simultaneous lesion referral that the AI deemed required dermatologist assessment they technically did have an in-person review before discharge. (b) Bar chart showing sensitivity of AI (95.3%) vs. dermatologists (88.5%; first diagnosis listed on surgical request form) confirmed by histopathological diagnosis. (c, d) Pie charts indicating (c) the percentage of correct vs. incorrect diagnosis or (d) pathological class of lesion [melanoma lineage, squamous cell carcinoma (SCC) lineage or basal cell carcinoma (BCC)]. McNemar’s test (b–d): **P < 0.01; ****P < 0.0001.

Of the included 1230 lesions assessed, 711 (57.8%) had no independent dermatologist review and a diagnosis was suggested by the AI, with the patient next seeing an in-person NHS dermatologist. A total of 519 (42.2%) AI assessments were reviewed by an independent dermatologist, before either discharge or referral to an in-person dermatologist. Some 274 of the initial 1230 lesions (22.3%) were discharged prior to assessment by an in-person dermatologist, representing appointments saved by the AI. A range of management plans were initiated by the in-person dermatologist, including discharge (which included treatment with topical medications or cryotherapy), further review or biopsy (including by alternate specialties, such as plastic surgery, if required). The outcomes of these referrals are shown in the Sankey diagram in Figure 2(a).

To assess the performance of the AI algorithm, we first examined the sensitivity, specificity, PPV and NPV in comparison with dermatologists. In line with prior findings,^19^ the AI algorithm is highly sensitive, with a sensitivity of 95.3% [95% confidence interval (CI) 90.5–98.1] (Figure 2b, Table 2). This compared favourably (*P* = 0.006) with dermatologists, with a sensitivity in the same lesions of 88.5% (95% CI 82.3–93.2; although, importantly, the dermatologist was suspicious enough to refer the lesion for excision or biopsy, even if this diagnosis was not listed as their most likely differential diagnosis).

**Table 2:** Summary of the estimates of the sensitivity, specificity, positive (PPV) and negative predictive value (NPV), and overall accuracy of artificial intelligence (AI) and dermatologists in our study

	AI	Dermatologists
Sensitivity (%)	95.3 (90.5–98.1)	88.5 (82.3– 93.2)
Specificity (%)	21 (15.6–27.2)	61 (53.9–67.7)
PPV (%)	46.5 (44.6–48.5)	62.1 (53.9–67.7)
NPV (%)	85 (74–93)	88 (82.3–92.1)
Histopathological accuracy (%)		
Precise diagnosis	28.6	61.6
Correct tumour/lesion type	51.4	75.5

Data in parentheses are the 95% confidence intervals.

Specificity was 21% (95% CI 15.6–27.2) for AI, compared with 61% (95% CI 53.9–67.7) for dermatologists (*P* < 0.001). The PPV of the AI algorithm was 46.5% (95% CI 44.6–48.5). This compared with 62.1% in dermatologists (95% CI 57.8–66.2). The NPV of AI was 85% (95% CI 74–93) compared with 88% (95% CI 82.3–92.1) in dermatologists (Table 2).

To directly assess the histopathology-confirmed accuracy of AI in comparison with dermatologists, we sought to investigate the results of the biopsies performed only in cases in which no human review occurred, as this represented the diagnosis that was presented to the clinician by the AI in the clinical record. Of the 711 patients with no independent dermatologist review prior to clinic appointment, 318 went on to have a biopsy. We assessed whether the AI diagnosis presented to the in-person clinician matched the precise final histopathological diagnosis in these patients. This was correct in 28.6% (*n* = 91/318) of cases. The dermatologist correctly identified the precise diagnosis 61.6% (*n* = 196/318) of the time, a statistically significant increase (*P* < 0.001; Figure 2c, Table 2). For comparison, of these 318 biopsies, there were 2 in which no diagnosis was mentioned on the GP referral, and the GP was correct in comparison to the final histopathology in 22.5% (*n* = 71/316) of cases.

To expand this analysis, we investigated whether AI showed an improved ability to diagnose the lineage of lesion (e.g. a diagnosis of Bowen disease that turned out to be cSCC, or a diagnosis of melanoma that turned out to be a benign or dysplastic naevus would both be scored as correct). AI did this 51.6% (*n* = 164/318) of the time, in comparison with dermatologists who did so in 75.5% (*n* = 240/318) of cases (*P* < 0.001; Figure 2d).

Recent policy allows for AI algorithms assessing skin cancer referrals within the NHS to function autonomously.^10^ As such, we sought to further analyse the potential value that the human review was adding. Of the 519 AI decisions receiving a human review, 208 were because the original image was unsuitable for analysis by the AI algorithm. Of the remaining 311 patients (in which the AI suggested a diagnosis based on the images), 231 were discharged by the independent review and 80 were sent for in-person assessment, 35 of whom ended up having biopsies. Four of these patients were deemed benign by the AI but were diagnosed with malignancy (one SCC and three BCCs), representing cancers that would have been missed with no independent dermatologist review, and a number consistent with prior studies.^21^ This suggests that, although the digital review of AI decisions is not picking up many additional skin cancers, the review process still results in the detection of cancers that may otherwise be missed.

Furthermore, of the 711 patients who underwent no human review prior to in-person dermatology clinic appointment, 3 patients who were diagnosed by AI as having likely AK lesions subsequently turned out to have malignancy (1 invasive malignant melanoma and 2 BCCs). Importantly, the output of the AI assessment did allow for these patients to be seen in clinic. However, in an alternative management pathway where these referrals may not be referred for in-person dermatology assessment, AKs may be deprioritized and only seen after significant delay. This would have the potential to increase the stage at tumour presentation which, especially in the case of melanoma and SCC, has a significant detrimental effect on patient outcomes.^22^

## Discussion

We performed an assessment of the real-world performance of AI in the diagnosis of skin cancer to provide crucial postimplementation data.^16^ We analysed the clinical pathways of the first 1230 lesions seen in our specialist clinics following AI assessment. We found that the AI algorithm had a high sensitivity in the detection of skin cancer. However, when compared with the final histopathological diagnosis, AI correctly presented this diagnosis to dermatologists in only 28.6% of cases.

There are several possible reasons to explain this phenomenon. Firstly, the real-world images being used in the patient pathway are likely to be of inferior quality compared with those used in the initial algorithm training. Secondly, and importantly, what is not visible to the dermatologist is the exact decision-making process behind each AI diagnosis. For example, the AI algorithm may consider a lesion to have a 95% likelihood of being a benign naevus, but a 5% chance of being a melanoma: if this lesion is ‘diagnosed’ by the AI as a melanoma as a means of increasing sensitivity, but turns out to be a benign naevus, then this is clearly a safer, if less accurate, approach, and carries with it a risk of overdiagnosis and overtreatment. Indeed, the algorithm is designed with this purpose in mind and can be altered based on each individual Trust’s preference for risk stratification.^19^

Recent data have suggested that the use of AI in clinical settings has the potential to negatively influence clinician decision-­making,^23^ termed ‘automation bias’. Given that the AI algorithm assessed here correctly identifies the precise histopathological diagnosis 28.6% of the time, incorrect diagnoses have the potential to influence the subsequent clinical assessment. Therefore, to improve the information provided to the dermatologist, the AI could instead classify lesions broadly, as likely ‘malignant’, ­‘premalignant’ or ‘benign’. This would prevent undue influence over the reviewing dermatologist and take advantage of the high sensitivity of the AI algorithm while avoiding the current pitfalls in its diagnostic ability.

Furthermore, our study highlights a concern with a recent policy decision that allows for AI to function autonomously in skin cancer screening, without human review.^10^ While we show the majority of benign diagnoses made by AI do not result in skin cancer diagnoses, seven were cancerous (including lesions defined as AKs; although how each Trust handles potential AKs is dependent on how they choose to triage the algorithm’s outputs): cancers that may have been missed in a situation in which AI acted autonomously. When patients were asked their opinion on the use of AI in skin cancer diagnosis and screening, the vast majority were in favour of it being used to assist clinician assessment, but only a minority were in favour of AI being used as a standalone, autonomous tool.^24^

Previous studies have suggested that optimal performance is reached when AI and clinicians are used in combination,^9,17,25^ and that double assessment of teledermatology referrals is more accurate than single review.^26^ This is consistent with our findings that human review of AI decision-making improves its sensitivity. Additionally, the benefits of AI working in collaboration with dermatologists are further highlighted by prior findings that dermatologists themselves have a small but present risk for misclassifying malignancies as benign.^27^ It is important to balance the risk of possible missed cancers against the fact that the AI algorithm facilitated increased capacity by allowing for discharge of approximately 20% of patients prior to dermatologist assessment. The health economics of this are outside the scope of this study.

It is important to state that the findings of this study represent the current state of play of AI in clinical use. One of the benefits of AI is that the algorithms can continue to improve based on prior experience. Data fed back into the algorithm should, in theory, increase its accuracy over time, improving its use as a valuable tool in skin cancer diagnosis. Our study suggests that the final histopathological diagnosis is what should be fed back into the algorithm, as opposed to clinician diagnosis, given dermatologists themselves were definitively correct an imperfect 61.6% of the time.

A shortcoming of our study is that it represents the experience of only one centre. Further research investigating the performance of AI algorithms in skin cancer diagnosis in multiple centres and countries will provide a fuller understanding of its real-world performance. An additional limitation is that the comparison of diagnostic accuracy between AI and humans is not truly ‘like-for-like’. Dermatologists were reviewing cases already triaged and reported by AI, creating a selection bias and representing a population not truly representative of the baseline population. However, as highlighted above, AI use has the potential to influence clinician performance,^23^ and thus the impact of this selection bias is unexplored and would be an interesting subject of future work.

We here present a comprehensive analysis of the diagnostic accuracy, compared with the final histopathological diagnosis, of AI employed on a large-scale, real-world cohort of patients assessed as part of the NHS skin cancer screening pathway. While undoubtedly AI has the potential to reduce clinical workloads and assist in lesion stratification, our data suggest that there remains significant room for improvement in the everyday diagnostic accuracy of AI algorithms employed in the NHS, which in turn would reduce the risk of automation bias in clinicians. Our study has important policy implications and suggests that it may be premature to entirely abolish human review of AI decision-making in skin cancer referrals.

## Supplementary Material

vzag068_Supplementary_Data
